# Expression of human milk fat globulin proteins in cells of haemopoietic origin

**DOI:** 10.1054/bjoc.2000.1404

**Published:** 2000-09-04

**Authors:** W Krüger, R Lohner, R Jung, N Kröger, A R Zander

**Affiliations:** Bone Marrow Transplantation Centre, Dept. Oncology/Haematology, Dept. Clinical Chemistry, University-Hospital Eppendorf, Martinistraße 52, Hamburg, 20246, Germany

**Keywords:** human milk fat globulin, mucins, breast cancer, lactadherin (BA46), BA70

## Abstract

Lineage-specific gene expression has been used for the identification of metastasis of cancers with unknown primary site or of disseminated cancer cells in haemopoietic compartments such as bone marrow or in lymph nodes. For the muc1, cytokeratin-19 and the CEA genes, the transcription in haemopoietic cells has been shown recently. Here, the expression of the mammary epithelium related antigens BA46 (lactadherin) and BA70 in lymphoid and myeloid cell lines, and in clinical specimens is analysed. By Northern-hybridization with specific oligonucleotides an ubiquitous transcription of both genes, independent from the provenance of cells or the chromosomal gender was found. Both mRNA molecules were amplified by rtPCR from the samples and the specificity could be confirmed by sequence analysis. Peptide-specific antibodies were raised in rabbits and used for Western-blot analysis and for immunocytochemical studies. Both antibodies reacted with total cell lysates from myeloid and lymphatic cells. In immunocytochemistry antibody P717 (anti-lactadherin) had a significant strong staining of the myeloid cell lines K562 and HL60 suggesting a participation of lactadherin in leukocyte-function. Using antibody P718, strong stains were seen in myeloid line K562 and lymphoid line ST486. In conclusion, our findings expand the results that the concept of lineage-specific gene expression is no longer valid at the molecular level. © 2000 Cancer Research Campaign


*British Journal of Cancer* (2000) **83**(7), 874–879
© 2000 Cancer Research Campaign
doi: 10.1054/ bjoc.2000.1404, available online at http://www.idealibrary.com on IDEAL®

### Expression of human milk fat globulin proteins in cells of haemopoietic origin

**W Krüger, R Lohner, R Jung*, N Kröger and AR Zander**

Bone Marrow Transplantation Centre, Dept. Oncology/Haematology, *Dept. Clinical Chemistry, University-Hospital Eppendorf, Martinistraße 52, 20246 Hamburg, Germany

**Summary** Lineage-specific gene expression has been used for the identification of metastasis of cancers with unknown primary site or of disseminated cancer cells in haemopoietic compartments such as bone marrow or in lymph nodes. For the muc1, cytokeratin-19 and the CEA genes, the transcription in haemopoietic cells has been shown recently. Here, the expression of the mammary epithelium related antigens BA46 (lactadherin) and BA70 in lymphoid and myeloid cell lines, and in clinical specimens is analysed. By Northern-hybridization with specific oligonucleotides an ubiquitous transcription of both genes, independent from the provenance of cells or the chromosomal gender was found. Both mRNA molecules were amplified by rtPCR from the samples and the specificity could be confirmed by sequence analysis. Peptide-specific antibodies were raised in rabbits and used for Western-blot analysis and for immunocytochemical studies. Both antibodies reacted with total cell lysates from myeloid and lymphatic cells. In immunocytochemistry antibody P717 (anti-lactadherin) had a significant strong staining of the myeloid cell lines K562 and HL60 suggesting a participation of lactadherin in leukocyte-function. Using antibody P718, strong stains were seen in myeloid line K562 and lymphoid line ST486. In conclusion, our findings expand the results that the concept of lineage-specific gene expression is no longer valid at the molecular level. © 2000 Cancer Research Campaign

**Keywords:** human milk fat globulin; mucins; breast cancer; lactadherin (BA46); BA70

The detection of so-called lineage-specific antigens has become a standard tool of pathologists for the identification of metastasis of cancers with unknown primary site or of disseminated cancer cells in haemopoietic compartments such as bone marrow or in lymph nodes (Pantel et al, 1994). Marker proteins used for the identification of epithelial cells are the cytokeratins and the mucins (Dearnaley et al, 1983). Whilst the cytokeratins have been well described as part of the cytoskeleton of epithelial cells the function of the mucins remains less clear. The mucins are a class of highly glycosylated proteins. The main interest in mucin-research was focused on their expression on epithelial cancer cells and their possible usefulness as tumour markers and targets for antineoplastic immunotherapy so far (Gendler and Spicer, 1995; Patton et al, 1995). The expression of the muc1 protein by haemopoietic cells has recently been described by two groups. Brugger et al have investigated its expression by FACS-analysis and Dent et al demonstrated muc1 transcription in blood cells by molecular methods (Brugger et al, 1999; Dent et al, 1999).

The luminal membrane of human breast epithelial cells contains a variety of mucin-like proteins, some of these named human milk fat globule proteins. These HMFG-proteins have been discussed as surface differentiation markers as well as proteins with anti-infectious function on milk vesicles. Two of these proteins have been characterized and cloned by Larocca et al. One protein has a molecular weight of 46 kD (BA46), the other is approximately 70 kD sized (BA70). Homologies with other mucin genes are neither described for the BA46 nor for the BA70 gene. Both proteins have been discussed as targets for immunotherapy and radioimaging of breast cancer (Larocca et al, 1990; 1991). The BA46 antigen has recently been renamed lactadherin. For lactadherin an anti-infectious effect protecting against rota-virus infections has been shown (Newburg, 1999). A weak transcription of both sequences has been detected in Raji cells, however, their expression neither in other haemopoietic cell lines nor in haemopoietic specimens from healthy female and male subjects has been considered so far.

Here we investigate the transcription and expression of BA46 and BA70 in haemopoietic cells by molecular and immunological approaches.

*Received 24 November 1999*
*Revised 12 June 2000*
*Accepted 28 June 2000*

*Correspondence to:* W Krüger

### MATERIAL AND METHODS

### Cell lines and clinical specimens

Breast cancer cell lines MCF-7, MDA-MB453, and haemopoietic lines ST486, HL60, K562, Raji, and Namalwa were purchased from the ATCC. Rat fibroblast line Rat-2 was used as negative control in immunocytochemistry. Cell lines were maintained in RPMI-1640 or MEM, supplemented with fetal calf sera and 1% L-glutamine at 37°C and 5% $CO_2$ according to the manufacturer's instructions following standard methods. Patients specimens consisting of bone marrow ($n$ = 11), G-CSF mobilized blood stem cell collections ($n$ = 3), and one pericardial effusion were obtained from healthy volunteers and from cancer patients after informed consent. All cell lines and clinical specimens investigated are listed in Table 1. Clinical samples were subjected to mononucleated cell separation by ficoll-centrifugation prior to analysis.

**Table 1** Cell lines and clinical samples

| Cell line/sample | Description | Diagnosis | Gender |
|---|---|---|---|
| MCF-7 | Cell line | Breast cancer | |
| MDA-MB453 | Cell line | Breast cancer | |
| K-562 | Cell line | CML | |
| HL-60 | Cell line | AML | |
| Raji | Cell line | NHL | |
| Namalwa | Cell line | NHL | |
| ST-486 | Cell line | NHL | |
| 1 | BM | BC | F |
| 2 | BM | VD | M |
| 3 | BM | VD | M |
| 4 | BM | VD | Not known |
| 5 | LP | VD | M |
| 6 | LP | BC | F |
| 7 | BM | VD | F |
| 8 | BM | VD | M |
| 9 | BM | VD | M |
| 10 | PE | LC | M |
| 11 | BM | CC | M |
| 12 | BM | VD | M |
| 13 | LP | VD | M |
| 14 | BM | VD | F |
| 15 | BM | VD | M |
| 16 | BM | VD | F |
| 17 | BM | VD | M |

Abbreviations: BM, bone marrow; LP, leukapheresis; CML, chronic myeloid leukaemia; AML, acute myeloid leukaemia; NHL, non-Hodgkin's lymphoma; BC, breast cancer; VD, healthy volunteer donor; LC, lung cancer; CC, colon cancer; M, male; F, female

### RNA-extraction and Northern blot analysis

RNA was extracted with guanidinium thiocyanate according the method from Sacchi and Chomczinsky. 15 µg of RNA were separated by electrophoresis at 50 V through 0.8% agarose gels. Nucleic acid fragments were stained with ethidium bromide for documentation. Capillary blot onto positively charged nylon membranes (Zeta-probe, BioRad, Munich, Germany) was performed under alkaline conditions. Hybridization assay was carried out with $\gamma$-$^{32}$P-labelled oligonucleotides complementary to the mRNA-sequence of the 46 kD and 70 kD milk fat globule proteins. Hybridization with an oligonucleotide complementary to the human β-actin mRNA served as positive and with an oligonucleotide identical (sense) to the BA46- and BA70-sequence as negative control (Kruger and Pulz, 1991; Duggan et al, 1997). The sequence as oligonucleotides for hybridization and PCR-amplification is shown in Table 2.

### Reverse transcriptase polymerase chain reaction and sequence analysis

One microgram of mRNA was subjected to reverse transcription with primers shown in Table 2. In general, reverse transcriptase polymerase chain reaction was performed as previously described. The house-keeping gene β-actin was amplified as positive control, $H_2O$ was used as negative control. Sequence analysis of amplified fragments was performed in an automated sequencer (Applied Biosystems). PCR-products were sequenced directly by the dideoxynucleotide chain termination method using fluorescent labels with dye terminator kits directly. Comparison of sequences obtained with the BA46 and BA70 mRNA was made with a software using a Needleman & Wunsch algorithm at the EMBO, Heidelberg, Germany (Kruger et al, 1996).

To ensure the specific amplification of mRNA-derived cDNA in PCR-experiments, cellular DNA extracted from cell lines MCF7 and Raji was subjected to amplification with milkfat-globuline-specific primers. The amplicons produced by this approach were significantly larger than those obtained from rtPCR with cDNA indicating the presence of introns in cellular DNA derived PCR-products.

### Antibodies

Two polyclonal antibodies against epitopes of the 46 kD and 70 kD milk fat globulin proteins were raised in rabbits grown under pathogen-free conditions. Peptides 717 (DFIHD-VNKKHKEFV, BA46) and 718 (RSKWSERTRKPLEALY, BA70) were synthesized according to the corresponding mRNA sequences of BA46 and BA70. Peptides were linked to keyhole limpet haemocyanin and two rabbits were immunized on days 0, 14, 28 and 56, each with a different peptide. Animals were bled and the specific reactivity of the sera was determined by enzyme-linked immunosorbent assay. Subsequently, polyclonal antibodies P717 and P718 were purified by immunoaffinity using peptides 717 and 718 as specific matrix components. Enriched polyclonal antibodies were used in Western-blot analysis and for immunocytochemistry.

### Immunocytochemistry

Cells subjected to immunocytochemistry were mobilized from culture flasks with cell scrapers to avoid trypsinization. Immunocytochemistry was carried out following standard protocols. After incubation with polyclonal antibodies P717 and P718, antibody-labelled cells were detected by a sheep-anti-rabbit

**Table 2** Oligionucleotides for Northern hybridization and rtPCR

| Oligonucleotide | Sequence | Localization |
|---|---|---|
| 46H | CACACATCACATTCCCATGGTGGCCTCAAG | 1021–1050 |
| 70H | CATCTGCCGGCACCTGCTGCCCCGGGTCCA | 126–155 |
| β-actin | CGGTTCCGCTGCCCTGAGGCACTCTTGCAG | 801–830 |
| NC46 | CTTGAGGCCACCATGGGAATGTGATGTGTG | 1021–1050 |
| NC70 | GATCCTTCTCACCGTGCACAATCAAGGCGG | 161–190 |
| 46PCRs | TAAGCCCCGTCCCCTAAC | 848–865 |
| 46PCRa | GCTGGGCTTCAGGACAAG | 1211–1194 |
| | Amplicon length | 364Bp |
| 70PCRs | AGCCTCTAGAAGCCCTCTATGG | 29–50 |
| 70PCRa | AGGTTGTGTTTGCCTTCTGG | 278–259 |
| | Amplicon length | 250Bp |

alkaline phosphatase conjugate (P717, P718) followed by an enzyme-mediated colour reaction. Primary antibodies were used in dilutions between 1:2 up to 1:1000. Positive cells and non-stained cells were counted and the percentage of stained cells was calculated according to Pantel et al (1994). The percentages of stained cells were compared by univariate analysis (least significance difference) using the computer software WinSTAT (Kalmia Co. Inc; Cambridge, USA).

### Western blot analysis

Eighty micrograms of total cellular protein were separated by SDS-polyacrylamide gel electrophoresis through 5%, 7.5%, 12.5% and 15% gels. This selection of acrylamide allowed separation of proteins between 10 and 200 kD molecular weight. All gels were prepared in duplicate. One gel of each run was subjected to silver stain, the other was blotted onto a nitrocellulose membrane. Immunoblot analysis was performed following standard protocols with antibodies P717 and P718 (Mohanam et al 1997).

### RESULTS

Transcription of the BA46 and BA70 messenger-RNA could be detected by specific oligonucleotide-hybridization in all cell lines and clinical samples investigated. Co-hybridization with the control oligonucleotide homologue to the sense-sequence of the BA46 mRNA remained negative in all cases (Table 3) (Figure 1).

Reverse transcriptase polymerase chain reaction with primers for the sequences of the BA46 and the BA70 proteins produced nucleic acid fragments of 364 bp and 270 bp in all cases investigated. Sequence analysis of PCR-products and comparison to the BA46 and BA70 mRNA sequences using a Needleman and Wunsch algorithm confirmed identity for all cell lines and for eight clinical samples investigated (Table 3). The result of sequence comparison for the BA70 sequence shows (Figure 2), data for the BA46 sequence are not shown.

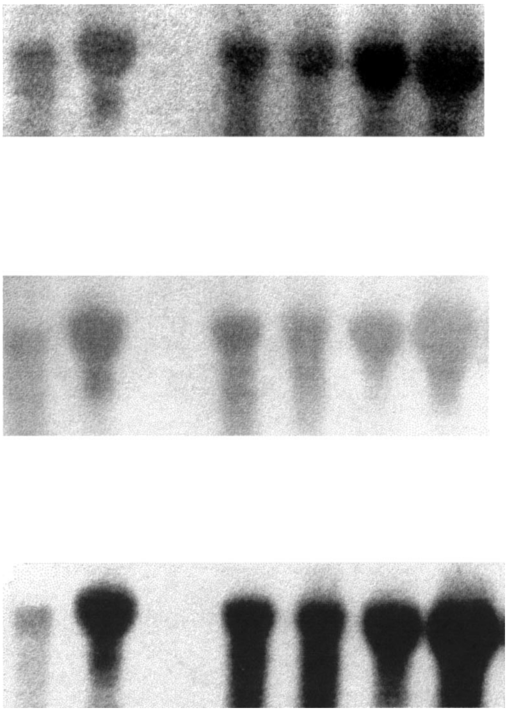

**Figure 1** Northern blot hybridization with β-actin- (top), BA46- (middle), and BA70- (bottom) oligonucleotides. Lanes: 1) MCF7, 2) K562, 3) $H_2O$, 4) Patient #9, 5) Patient #3, 6) ST486, 7) HL60.

**Table 3** Results of Northern-blot, reverse transcriptase polymerase chain reaction, and sequence analysis

| Specimen | 46H | 70H | β-actin | NC 46 | NC 70 | 46PCR | 46seq | 70PCR | 70seq |
|---|---|---|---|---|---|---|---|---|---|
| MCF-7 | + | + | + | – | – | + | + | + | + |
| MDA-MB453 | + | + | + | – | – | + | + | + | + |
| K-562 | + | + | + | – | – | + | + | + | + |
| HL-60 | + | + | + | – | – | + | + | + | + |
| Raji | + | + | + | – | – | + | + | + | + |
| Namalwa | + | + | + | – | – | + | + | + | + |
| ST-486 | + | + | + | – | – | + | + | + | + |
| 1 | + | + | + | | | | | | |
| 2 | + | | + | | | | | | |
| 3 | + | + | + | | | + | + | + | + |
| 4 | + | + | + | | | | | | |
| 5 | + | + | + | | | | | | |
| 6 | + | | + | | | | | | |
| 7 | + | + | + | | | + | + | + | + |
| 8 | + | | + | | | | | | |
| 9 | + | + | + | | | | | | |
| 10 | + | | + | | | | | | |
| 11 | + | | + | | | | | | |
| 12 | | | | | | + | + | + | + |
| 13 | | | | | | + | + | + | + |
| 14 | | | | | | + | + | + | + |
| 15 | | | | | | + | + | | |
| 16 | | + | + | | | | | + | + |
| 17 | | + | + | | | | | + | + |

+, positive; –, negative; empty, not done

*© 2000 Cancer Research Campaign*

**Figure 2** Nucleic acid sequences of the BA70 mRNA compared with sequences of amplicons obtained by rtPCR with primer pairs 70PCRs/70PCRa

The results of immunological experiments with polyclonal antibodies P717 and P718 were divergent for Western blot analysis and for immunocytochemistry. Antibody P717 showed in Western blots a strong reaction with a protein of approximately 30 kD molecular weight. This protein was detected in breast cancer cells, lymphatic and myeloid cells. The expression seems to be strongest in cell lines MCF7, MDA-MB453, K562, and Raji. Furthermore, an additional reaction was seen in the range of 80 kD molecular mass. The intensity of reaction varied between the cells, however, the reaction was strongest in breast cancer cell extracts (Figure 3).

Using antibody P718, a strong reaction with a protein of 28 kD molecular mass was detected in total protein extracted from breast cancer cell line MCF7 and MDA-MB453. A second protein in the range of 85 kD was detected in lysates from all cell lines independent from their provenance (Figure 3).

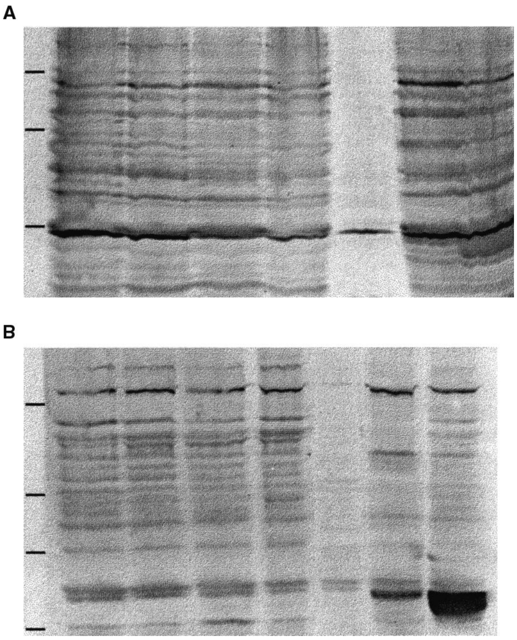

**Figure 3** Western blot analysis with antibodies P717 (**A**) and P718 (**B**) **A**) Western blot with P717, from left to the right: molecular mass symbols (84 kD, 60 kD, 30.5 kD), K562, Raji, Namalwa, ST486, HL60, MDA-MB453, MCF7 **B**) Western blot with P718, from left to the right: molecular mass symbols (84 kD, 48.5 kD, 30.5 kD, 26.5 kD), Raji, Namalwa, K562, ST486, HL60, MDA-MB453, MCF7

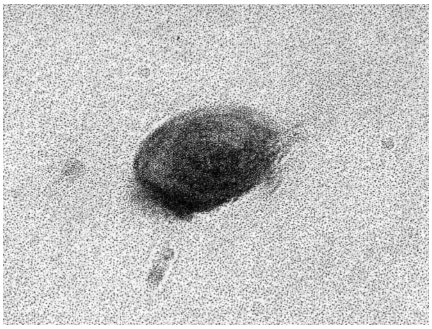

**Figure 4** Staining of an MCF7 cell with polyclonal antibody P717

Subsequently, antibodies P717 and P718 were used for immunocytochemical investigations. Differences in the pattern of reactions were seen for both antibodies. The reaction of P717 with breast cancer cells was weaker than expected. The staining pattern of MCF7 cells is shown in Figure 4. Preferably parts of the apical membrane were stained, however, in total ca. 15% of cells were stained. Lymphatic cells were hardly detected by P717. Reaction was strongest with the myeloid cell line K562 followed by breast cancer line MCF7 and myeloid line HL60. For K562 the differences were significant ($P = 0.02$) in univariate analysis to all other cells investigated. Data are presented graphically in Figure 6.

Pattern of reaction with P718 was different with generally lower staining rates. Highest rates were again seen for myeloid line K562 followed by lymphoid line ST486 and breast cancer cell line MCF7 (Figure 5). In this assay reaction with myeloid line HL60 and lymphoid line Namalwa was weak or absent, respectively (Figure 6). Burkitt-lymphoma derived cell line Raji was neither detected by P717 nor by P718.

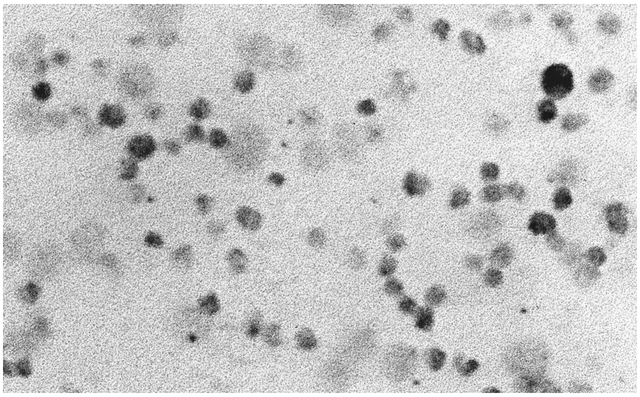

**Figure 5** Staining of ST486 cells with polyclonal antibody P718

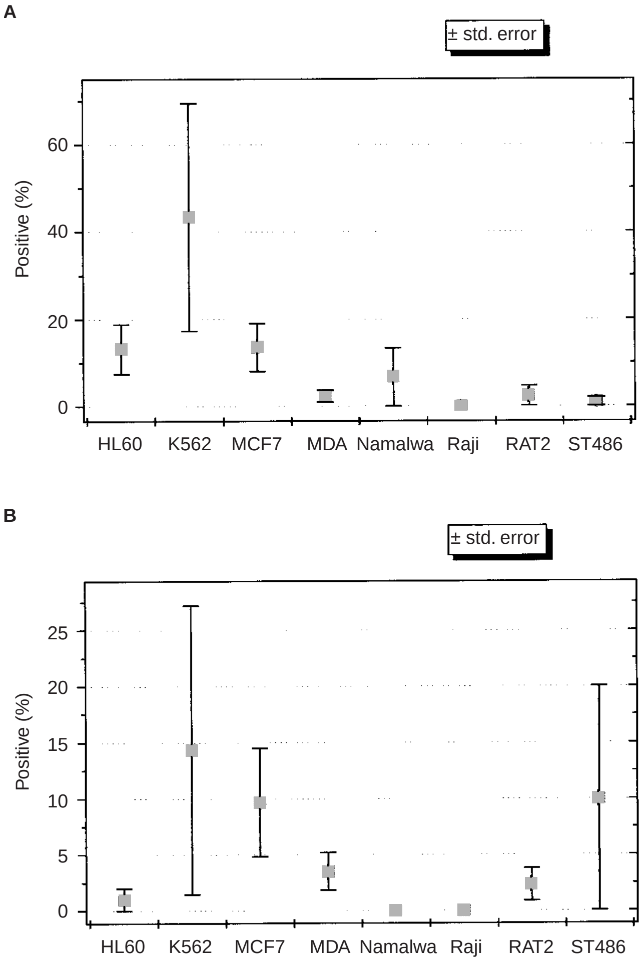

**Figure 6** Labelling of cells with polyclonal antibodies P717 (**A**) and P718 (**A**). Shown is the percentage of stained cells (MDA: MDA-MB453)

### DISCUSSION

The expression of the proteins BA46 and BA70, which are expressed constitutively in the mammary epithelium was studied in haemopoietic cell lines and tissue extracts were studied by molecular and immunological methods. On the molecular level we could clearly demonstrate that both messenger RNAs are ubiquitously transcribed in myeloid and lymphatic cancer cell lines and in bone marrow and blood stem cell samples obtained from

 *© 2000 Cancer Research Campaign*

healthy volunteers and from patients with epithelial cancer. For two reasons, the transcription of the BA46 and BA70 in myeloid and lymphoid cell lines can not be regarded as phenomenon of gene-dysregulation due to malignant transformation. First, two myeloid lines of different stage of differentiation and three lymphatic lines were included in the experiments. Differences were not seen on the molecular level arguing for a constitutive expression rather then a regulatory one. Second, results from marrow and stem cell samples included from volunteers clearly demonstrate that the transcription in haemopoietic tissue is ubiquitous and not restricted to tumour cells. Larocca et al described an approximately 47% homology of the BA46 antigen to the blood clotting factors V and VII (Larocca et al, 1991). Extended homologies of the BA70 have not been described so far (Larocca et al, 1990). We could confirm the specificity of our rtPCR assays by sequence analysis and sequence alignment in all cases performed. Our samples included some specimens from patients with epithelial cancer. Here, it could be argued that sequences could have been amplified from disseminated cancer cells. However, results of hybridization assays clearly exclude this possibility of interference.

To immunolocalize BA46 and BA70 a new approach using antibodies against peptide-specific domains was performed. This approach was chosen since monoclonal antibodies have been made against both antigens, however, native BA46 and BA70 antigens are highly glycosylated (Larocca et al, 1990, 1991). Carbohydrates often alter the antigenicity of glycoproteins, thus a non-reactivity of monoclonal antibodies with non-glycosylated intracellular precursors could not be excluded. The ubiquitous high-level transcription of BA46 and BA70 specific messenger RNA suggests the synthesis of the proteins, at least as a non-glycosylated precursor molecule. Therefore, our approach was to investigate the protein expression first by polyclonal peptide-specific antibodies. Epitopes similar to those used were not found in protein databases so far. By this way, we could detect a significant reactivity of polyclonal antibodies P717 and P718 with total cell proteins from breast cancer cells, myeloid and lymphoid cells.

The pattern of reactivity of both antibodies in immunocytochemistry was different. BA46-derived antibody P717 showed a strong reactivity with myeloid cell lines K562 and HL60. The reactivity with lymphoid cells was weak or absent. In contrast, P718 bounded likewise strongest to K562 cells followed by MCF7 and lymphoid line ST486. These differences in staining patterns suggest at least differences in post-translational modifications such as degree of glycosylation, and furthermore a function of both proteins in haemopoietic cells. These results are hints that BA46 (lactadherin) participates in the non-specific anti-infectious function of myeloid cells (Newburg, 1999).

We conclude that mRNA of breast related antigens BA46 and BA70 antigens is transcribed in haemopoietic cells independent from their lineage, their state of differentiation, and their chromosomal gender. These findings argue for a vital role of these two proteins in normal haemopoietic cells, however, to define their physiological function further intensive research is necessary.

This study is corroborating earlier investigations concerning the muc1, cytokeratins or carcinoembryonic antigen which clearly show that the concept of lineage specific gene expression is at least on the molecular no longer valid (Jung et al, 1998, 1999; Dent et al, 1999).

### ACKNOWLEDGEMENTS

We wish to thank Dr Udo Schumacher, Dept Neuroanatomy, for critical reading of the manuscript and the Deutsche Krebshilfe for the support of the clinical trial.

### REFERENCES

Brugger W, Buhring HJ, Grünebach F, Vogel W, Kaul S, Müller R, Brummendorf TH, Ziegler BL, Rappold I, Brossart P, Scheding S and Kanz L (1999) Expression of MUC-1 epitopes on normal bone marrow: implications for the detection of micrometastatic tumor cells. *J Clin Oncol* **17**: 1535–1544

Dearnaley DP, Ormerod MG, Sloane JP, Lumley H, Imrie S, Jones M, Coombes RC and Neville AM (1983) Detection of isolated mammary carcinoma cells in marrow of patients with primary breast cancer. *J R Soc Med* **76**: 359–364

Dent GA, Civalier CJ, Brecher ME and Bentley SA (1999) MUC1 expression in hematopoietic tissues. *Am J Clin Pathol* **111**: 741–747

Duggan C, Kennedy S, Kramer MD, Barnes C, Elvin P, McDermott E, O'Higgins N and Duffy MJ (1997) Plasminogen activator inhibitor type 2 in breast cancer. *Br J Cancer* **76**: 622–627

Gendler SJ and Spicer AP (1995) Epithelial mucin genes. *Annu Rev Physiol* **57**: 607–634

Jung R, Krüger W, Hosch S, Holweg M, Kröger N, Gutensohn K, Wagener C, Neumaier M and Zander AR (1998) Specificity of reverse transcriptase polymerase chain reaction assays designed for the detection of circulating cancer cells is influenced by cytokines in vivo and in vitro. *Br J Cancer* **78**: 1194–1198

Jung R, Petersen K, Krüger W, Wolf M, Wagener C, Zander A and Neumaier M (1999) Detection of micrometastasis by cytokeratin 20 RT-PCR is limited due to stable background transcription in granulocytes. *Br J Cancer* **81**: 870–873

Krüger W, Krzizanowski C, Holweg M, Stockschläder M, Kröger N, Jung R, Mross K, Jonat W and Zander AR (1996) Reverse transcriptase/polymerase chain reaction detection of cytokeratin-19 mRNA in bone marrow and blood of breast cancer patients. *J Cancer Res Clin Oncol* **122**: 679–686

Krüger WH and Pulz M (1991) Detection of *Borrelia burgdorferi* in cerebrospinal fluid by the polymerase chain reaction. *J Med Microbiol* **35**: 98–102

Larocca D, Peterson JA, Walkup G, Urrea R and Ceriani RL (1990) Cloning and sequencing of a complementary DNA encoding a Mr 70,000 human breast epithelial mucin-associated antigen. *Cancer Res* **50**: 5925–5930

Larocca D, Peterson JA, Urrea R, Kuniyoshi J, Bistrain AM and Ceriani RL (1991) A Mr 46,000 human milk fat globule protein that is highly expressed in human breast tumors contains factor VIII-like domains. *Cancer Res* **51**: 4994–4998

Mohanam S, Chintala SK, Go Y, Bhattacharya A, Venkaiah B, Boyd D, Gokaslan ZL, Sawaya R and Rao JS (1997) In vitro inhibition of human glioblastoma cell line invasiveness by antisense uPA receptor. *Oncogene* **14**: 1351–1359

Newburg DS (1999) Human milk glycoconjugates that inhibit pathogens. *Curr Med Chem* **6**: 117–127

Pantel K, Schlimok G, Angstwurm M, Weckermann D, Schmaus W, Gath H, Passlick B, Izbicki JR and Riethmuller G (1994) Methodological analysis of immunocytochemical screening for disseminated epithelial tumor cells in bone marrow. *J Hematother* **3**: 165–173

Patton S, Gendler SJ and Spicer AP (1995) The epithelial mucin, MUC1, of milk, mammary gland and other tissues. *Biochem Biophys Acta* **1241**: 407–423

© 2000 Cancer Research Campaign
